# Clonal Hematopoiesis and Cardiovascular Disease in Patients With Multiple Myeloma Undergoing Hematopoietic Cell Transplant

**DOI:** 10.1001/jamacardio.2023.4105

**Published:** 2023-11-08

**Authors:** June-Wha Rhee, Raju Pillai, Tianhui He, Alysia Bosworth, Sitong Chen, Liezl Atencio, Artem Oganesyan, Kelly Peng, Tati Guzman, Kara Lukas, Brianna Sigala, Aleksi Iukuridze, Lanie Lindenfeld, Faizi Jamal, Pradeep Natarajan, Scott Goldsmith, Amrita Krishnan, Michael Rosenzweig, F. Lennie Wong, Stephen J. Forman, Saro Armenian

**Affiliations:** 1 Department of Medicine, City of Hope Comprehensive Cancer Center, Duarte, California; 2 Department of Pathology, City of Hope Comprehensive Cancer Center, Duarte, California; 3 Department of Population Sciences, City of Hope Comprehensive Cancer Center, Duarte California; 4 Cardiovascular Research Center and Center for Genomic Medicine, Massachusetts General Hospital, Boston; 5 Department of Hematology & Hematopoietic Transplantation, City of Hope Comprehensive Cancer Center, Duarte, California; 6 Department of Pediatrics, City of Hope Comprehensive Cancer Center, Duarte, California; 7 Department of Medicine, Harvard Medical School, Boston, Massachusetts

## Abstract

**Importance:**

There is a paucity of information on the association between clonal hematopoiesis of indeterminate potential (CHIP) and cardiovascular disease (CVD) in patients with cancer, including those with multiple myeloma (MM) undergoing hematopoietic cell transplant (HCT), a population at high risk of developing CVD after HCT.

**Objective:**

To examine the association between CHIP and CVD in patients with MM and to describe modifiers of CVD risk among those with CHIP.

**Design, Setting, and Participants:**

This was a retrospective cohort study of patients with MM who underwent HCT between 2010 and 2016 at City of Hope Comprehensive Cancer Center in Duarte, California, and had pre-HCT mobilized peripheral blood stem cell (PBSC) products cryopreserved and accessible for CHIP analyses. The study team performed targeted panel DNA sequencing to detect the presence of CHIP (variant allele frequency 2% or more).

**Main Outcomes and Measures:**

The primary end point was the 5-year cumulative incidence and risk for developing de novo CVD (heart failure, coronary artery disease, or stroke) after HCT.

**Results:**

Of 1036 consecutive patients with MM (580 male [56%]; median age, 60.0 years) who underwent a first autologous HCT, 201 patients had at least 1 CHIP variant (19.4%) and 35 patients had 2 or more variants (3.4%). The 5-year incidence of CVD was significantly higher in patients with CHIP (21.1% vs 8.4%; *P* < .001) compared with those without CHIP; the 5-year incidence among those with 2 or more variants was 25.6%. In the multivariable model, CHIP was associated with increased risk of CVD (hazard ratio [HR], 2.72; 95% CI, 1.70-4.39), as well as of individual outcomes of interest, including heart failure (HR, 4.02; 95% CI, 2.32-6.98), coronary artery disease (HR, 2.22; 95% CI, 1.06-4.63), and stroke (HR, 3.02; 95% CI, 1.07-8.52). Patients who had both CHIP and preexisting hypertension or dyslipidemia were at nearly 7-fold and 4-fold increased risk of CVD, respectively (reference: no CHIP, no hypertension, or dyslipidemia).

**Conclusion and Relevance:**

CHIP was significantly and independently associated with risk of CVD in patients with MM undergoing HCT and may serve as a novel biologically plausible biomarker for CVD in this cohort. Patients with MM and both CHIP and cardiovascular risk factors had an exceptionally high risk of CVD. Additional studies are warranted to determine if cardiovascular preventive measures can reduce CHIP-associated CVD risk.

## Key Points

**Question:**

Is clonal hematopoiesis of indeterminate potential (CHIP) detected at the time of hematopoietic stem transplant (HCT) associated with increased rates of cardiovascular disease (CVD) among patients with multiple myeloma (MM) following HCT?

**Finding:**

In this cohort study of patients with MM undergoing HCT, CHIP was highly prevalent at baseline and associated with a significantly increased risk of CVD after HCT. Among patients with MM and CHIP, the 5-year incidence of CVD post-HCT was 21.1% and exceeded 30% in those who had both CHIP and a modifiable cardiovascular risk factor.

**Meaning:**

Pre-HCT CHIP may serve as a novel biomarker for CVD in patients with MM undergoing HCT.

## Teaser

This study examines the association between clonal hematopoiesis of indeterminate potential and cardiovascular disease in patients with multiple myeloma.

## Introduction

Autologous hematopoietic cell transplant (HCT) is an effective treatment for patients with multiple myeloma (MM), especially for individuals who are medically fit and demonstrate disease response to induction therapy.^1,2^ In fact, MM is the most common indication for autologous HCT, with more than 7000 patients undergoing transplant annually in the US alone.^3^ Research into survivorship issues during the past 2 decades has highlighted the high risk for developing cardiovascular disease (CVD) following autologous HCT, especially among patients with lymphoma attributed to pre-HCT exposures to cardiotoxic chemotherapies, HCT-associated conditioning exposures, and de novo modifiable cardiovascular risk factors (eg, hypertension, diabetes, dyslipidemia) that emerge after HCT.^4,5,6^ Yet, there is a paucity of information on the risk of CVD after HCT^7^ for patients with MM, including the role of emerging biologic modifiers of CVD risk in the general population.

Clonal hematopoiesis of indeterminate potential (CHIP) refers to clonal expansion of blood cells driven by somatic leukemogenic variants in otherwise healthy or patients or individuals without leukemia.^8^ In nononcology populations, CHIP has been associated with aging-related health conditions, including coronary artery disease (CAD),^9,10^ heart failure,^11^ and stroke.^12^ Accumulating data suggest CHIP is more prevalent in patients with cancer after cancer treatment exposures compared with age-matched controls, attributed in part to clonal selection that results from myelotoxic stressors.^13^

Despite the growing body of literature on the risk of CVD associated with CHIP in nononcology populations, there is limited information on the association between CHIP and CVD risk among patients with cancer, a population with high rates of CHIP that may be driven by the cancer itself, associated comorbidities, or cancer treatment exposures. Therefore, we examined the association between pre-HCT CHIP and CVD after HCT in a demographically diverse population of patients with MM and further explored the interaction between modifiable cardiovascular risk factors at the time of HCT and CHIP on long-term cardiovascular outcomes.

## Methods

### Study Population and Clinical Variables

This was a retrospective cohort of patients with MM who underwent a first autologous HCT at City of Hope Comprehensive Cancer Center in Duarte, California, between 2010 and 2016 and had mobilized peripheral blood stem cell (PBSC) products cryopreserved and accessible for CHIP analyses (1036 of 1047 [99%]). Demographic data (age at HCT, sex, race, and ethnicity), cancer diagnosis, variables necessary to derive the pretransplant HCT-comorbidity index (HCT-CI), Karnofsky performance score, and HCT details (eg, conditioning, PBSC mobilization regimen, and PBSC cluster of differentiation [CD] 34 with cell counts cells per kg or more less than 3 × 10^6^ per kg]) were abstracted from medical records. Height and weight at the time of HCT were used to derive patient body mass index (BMI). Information related to pre-HCT induction therapy was abstracted in a subset of patients (n = 666) with available pharmacy data; there were no differences in demographic and disease-related characteristics between those with and without induction therapy information. All patients included in the cohort received high-dose melphalan conditioning (standard dose, 200 mg/m^2^) with appropriate dose-reduction (140 mg/m^2^) in those with physiologic impairment (eg, creatinine clearance more than 40 mL per minute), per standard of care. Chronic health conditions that could increase the risk of CVD, including hypertension, diabetes, and dyslipidemia, were captured if they were documented in the medical record by their treating physician and/or if a patient was receiving medications for their management.^6^ High HCT-CI was defined as 3 or higher.^14^ Patients were considered to be in complete remission at HCT prior to conditioning if bone marrow plasma cells were less than 5%.^15^ Information on vital status and cause of death was obtained from the National Death Index and medical records.

Cardiovascular outcomes of interest included heart failure, CAD, or stroke. Heart failure was defined as new diagnosis of heart failure or associated diagnosis (eg, left ventricular dysfunction) along with new left ventricular ejection fraction decrease to less than 50% by echocardiogram and/or clinical evidence of heart failure (eg, dyspnea on exertion, lower extremity edema).^16^ CAD was defined as new diagnosis of myocardial infarction, acute coronary syndrome, or atherosclerotic heart disease and documentation consistent with the American College of Cardiology case definition and data standards including symptoms, electrocardiogram, and cardiac biomarkers changes.^17^ Stroke was defined as new clinical diagnosis of stroke, cerebrovascular accident, transient ischemic attacks, and any other neurologic compromise attributable to central nervous system injury with correlating imaging studies and clinical examination.^18^

### Sample Preparation and Next-Generation Sequencing

DNA was isolated with QIAamp DNA Mini Kit (Qiagen) from cryopreserved PBSC samples. We performed targeted exome sequencing of the extracted DNA using a custom-designed QIAseq amplicon-based panel of 108 CHIP-associated genes (Qiagen; list of genes available in eTable 1 in Supplement 1). DNA library preparation was conducted by polymerase chain reaction multiplex amplification through the City of Hope Clinical Cancer Genomics laboratory. The library quality and size distribution were determined using the Agilent 2100 Bioanalyzer system. Pair-end sequencing at 1000 × coverage depth was performed through the City of Hope Integrative Genomics Core on NovaSeq S4 flow cell with the average target coverage read depth of 560 times; 1395 of 1398 targets were covered over 1000 × read depth (99.8%).

### Variant Calling and Annotation

Alignment of sequence reads to the human genome (GRCh37/hg19), variant calling, and annotation were performed independently using 2 software applications—CLC Genomics Workbench 22.0 (CLCBio) and NextGENE 2.4 (Softgenetics). We adopted established classification guidelines for CHIP,^19^ including Catalog of Somatic Mutations in Cancer CHIP pathogenic variant calling guidelines and referenced previously published prespecified lists of CHIP variants (eTable 1 in Supplement 1).^20,21^ Variant allele frequency (VAF) 2% or more was used to define CHIP.^22,23^

### Statistical Analysis

Univariate analyses were performed to compare patient demographics, Karnofsky performance score, HCT-CI, induction therapy, PBSC cell count, disease status at HCT, and baseline cardiovascular risk factors between patients with and without CHIP at HCT, using 2-sided χ^2^ for categorical variables or 2-sample *t* tests (comparison of means) or Wilcoxon test (comparison of medians) for continuous variables. Multivariable logistic regression was used to identify factors independently associated with the odds ratio (OR) of having CHIP. Variables included in the regression model were those with a *P* value <.10 in the univariate analyses.

The primary outcome measure was cumulative incidence of CVD (heart failure, CAD, or stroke) by 5-year post-HCT. Time to first CVD event was computed starting from the date of HCT to the earliest date of disease (heart failure, CAD, or stroke) onset, date last known to be alive, receipt of a second HCT, date of death, or 5 years after HCT, whichever came first. All-cause mortality was treated as a competing risk. Patients alive beyond 5 years after HCT were censored at 5 years. Separate sensitivity analyses were performed censoring follow-up at the time of relapse. We also examined outcomes according to individual CVD subtypes. Patients with a history of CVD prior to HCT, determined by medical record review (same definitions as post-HCT CVD), were excluded from the analysis. We also performed exploratory analyses evaluating the association between specific somatic mutations and subsequent CVD incidence.

Univariable and multivariable analyses were conducted using the Fine-Gray subdistribution hazard model, calculating subdistribution hazard ratios (sHRs) and their 95% CIs to quantify magnitude of risk for developing CVD.^24^ To develop the multivariable model, we first examined the association between baseline variables at HCT and 5-year CVD in univariable analysis; variables with a *P *value <.10 were included in the multivariable model. The final multivariable model included age, race and ethnicity, HCT-CI, hypertension, diabetes, dyslipidemia, and CHIP status.

To understand the interaction between individual modifiable pre-HCT cardiovascular risk factors (hypertension, diabetes, dyslipidemia), CHIP, and subsequent CVD risk, we created separate models that allowed us to evaluate the differential risk of CVD in the following categories: (1) no pre-HCT cardiovascular risk factors and no CHIP (reference group), (2) pre-HCT cardiovascular risk factors and no CHIP, (3) no pre-HCT cardiovascular risk factors and CHIP, and (4) cardiovascular risk factors and CHIP. The cumulative incidence of CVD by each category was calculated as described above and multivariable regression analyses were performed to adjust for potential confounders.

All statistical analyses were 2-sided and a *P* value <.05 was considered statistically significant. SAS version 9.4 (SAS Institute) was used.

## Results

### Patient Characteristics

Table 1 summarizes demographic and clinical characteristics of the cohort. Median age at HCT was 60.0 (range, 34.9-77.1) years and most were male (56.0%), overweight/obese by BMI (68.8%), and had a high (3 or higher) HCT-CI score (47.4%). The race and ethnicity composition was 10.9% Asian, 15.3% Black, 25.0% Hispanic, 47.0% non-Hispanic White, and 1.8% other.

**Table 1.** Demographic and Clinical Characteristics of Patients With Hematopoietic Cell Transplant (HCT)

Characteristic	Total (n = 1036)	CHIP (n = 201)	No CHIP (n = 835)	*P* value
Age at HCT, median (range), y	60.0 (34.9-77.1)	63.0 (34.9-77.1)	58.6 (30.4-75.3)	<.001
Age at HCT quartile categories, y				
Q1 (30.4-54.4)	254 (24.5)	30 (14.9)	234 (26.8)	<.001
Q2 (54.4-59.9)	258 (24.9)	38 (18.9)	220 (26.4)	
Q3 (59.9-65.5)	263 (25.3)	67 (33.3)	196 (23.5)	
Q4 (65.5-77.1)	261 (25.2)	66 (32.8)	195 (23.4)	
Sex, No. (%)				
Female	456 (44.0)	88 (43.8)	368 (44.1)	.94
Male	580 (56.0)	113 (56.2)	467 (55.9)	
Race and ethnicity,^a^ No. (%)				
Asian	113 (10.9)	29 (14.4)	84 (10.1)	.002
Black	158 (15.3)	24 (11.9)	134 (16.1)	
Hispanic	259 (25.0)	33 (16.4)	226 (27.1)	
Non-Hispanic White	487 (47.0)	109 (54.2)	378 (45.3)	
Other^b^	19 (1.8)	6 (3.0)	13 (1.6)	
BMI^c^				
Underweight/healthy (<25)	277 (26.7)	59 (29.4)	218 (26.1)	.35
Overweight/obese (≥25)	759 (68.8)	142 (70.6)	617 (73.9)	
Median HCT-CI score (range)	3 (0-11)	3 (0-11)	2 (0-9)	.001
HCT-CI score				
0	209 (20.2)	26 (12.9)	183 (21.9)	.01
1-2	333 (32.1)	67 (33.3)	266 (31.9)	
≥3	494 (47.4)	108 (53.7)	386 (46.2)	
KPS, median (range)	90 (40-100)	90 (50-100)	90 (40-100)	.44
PBSC mobilization regimen, No. (%)				
G-CSF+ cyclophosphamide	868 (83.8)	169 (84.1)	699 (83.7)	.92
G-CSF+ plerixafor	194 (18.7)	41 (20.4)	153 (18.3)	.75
PBSC CD34+ count, median (range), 10^6^ cells/kg	7.5 (2.0-71.7)	7.0 (2.0-32.7)	7.5 (2.0-71.7)	.16
PBSC CD34+ count				
>3 × 10^6^ cells/kg	998 (96.3)	192 (95.5)	806 (96.5)	.51
≤3 × 10^6^ cells/kg	38 (3.7)	9 (4.5)	29 (3.5)	
Remission status at HCT, No. (%)				
Complete remission	170 (16.4)	21 (10.5)	149 (17.8)	.01
Not in complete remission	866 (83.6)	180 (89.6)	686 (82.2)	
Baseline cardiovascular risk factors, No. (%)				
Hypertension	587 (56.7)	109 (54.2)	478 (57.3)	.44
Diabetes	209 (20.2)	53 (26.4)	156 (18.7)	.02
Dyslipidemia	439 (42.4)	93 (46.3)	346 (41.4)	.22

Abbreviations: BMI, body mass index; CHIP, clonal hematopoiesis of indeterminate potential; CD34, cluster of differentiation 34; G-CSF, granulocyte colony-stimulating factor; HCT-CI, hematopoietic cell transplant-comorbidity index; KPS, Karnofsky performance scale; PBSC, peripheral blood stem cells.

^a^ Race and ethnicity were self-reported.

^b^ Includes Alaskan Native/American Indian, Hawaiian Native/Pacific Islander, mixed race, and other.

^c^ Calculated as weight in kilograms divided by height in meters squared.

**Figure 1.**
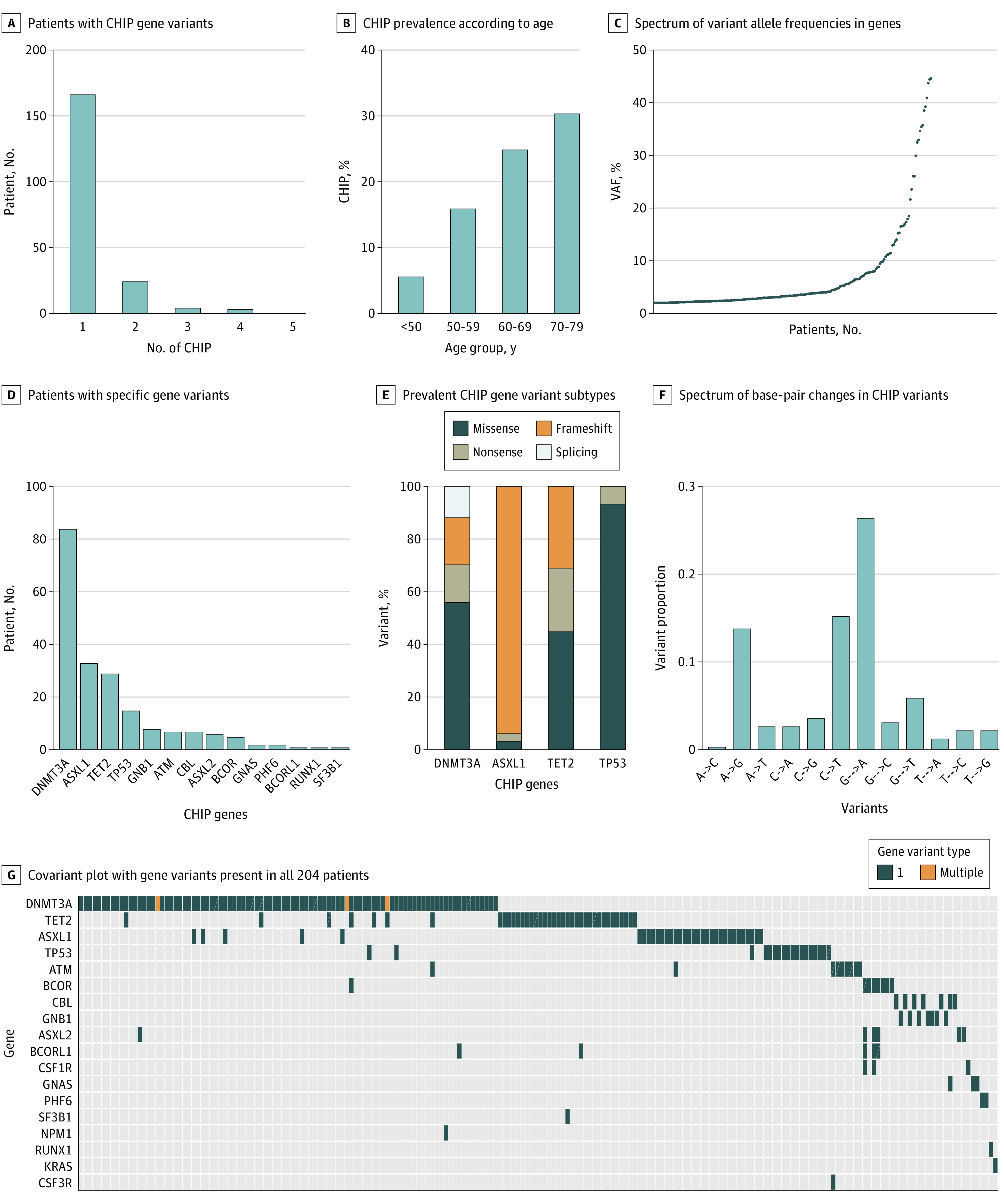
Characteristics of Clonal Hematopoiesis of Indeterminate Potential (CHIP) Mutations A, Number of patients harboring CHIP mutations in 1, 2, and 3 or more different genes; B, prevalence of CHIP according to age at transplant; C, spectrum of variant allele frequencies in genes: each dot represents an individual patient; D, number of patients with specific gene mutations; E, percentage of the different mutation subtypes for 4 of the most prevalent CHIP genes; F, spectrum of base-pair changes in the CHIP variants, G, comutation plot showing mutations present in all 204 patients: each column represents a single patient, variant allele frequency (VAF) cutoff used to identify mutations was 0.02.

Overall, 201 patients had at least 1 CHIP variant (19.4%) and 35 patients had 2 or more variants (3.4%) (Figure 1A; eTable 2 in Supplement 1). There was an increase in the prevalence of CHIP by age: older than 50 years, 5.8%; 50 to 59 years, 16.1%; 60 to 69 years, 25.1%; and older than 70 years, 30.5% (Figure 1B). Among patients with CHIP, the calculated VAFs were 2% to 5% in 135 patients (67.1%), 5% to 10% in 32 patients (15.9%), and 10% to 20% in 19 patients (9.4%) (Figure 1C). *DNMT3A* was the most frequently mutated gene (41.8%; R882 hot-spot mutations [9.5%]); other commonly mutated genes included *ASXL1* (16.4%; majority frameshift mutations), *TET2* (14.4%), and *TP53* (7.5%; majority missense mutations) (Figure 1D). The gene-specific variant frequency and functional categories and spectrum of base-pair changes of variants across genes are summarized in Figure 1E-G.

Compared with patients without CHIP, those with CHIP were significantly more likely to be older at HCT (median age, 63.0 vs 58.6 years; *P* < .001), have a high HCT-CI score (53.7% vs 46.2%; *P* = .01), less likely to be in complete remission (10.5% vs 17.8%; *P* = .01), and more likely to have diabetes (26.4% vs 18.7%; *P* = .02) (Table 1). There were no significant differences in induction treatments between the 2 groups (eTable 3 in Supplement 1) or by PBSC mobilization approaches and PBSC CD34+ count. In the multivariable model, older age at HCT (OR, 1.05; 95% CI, 1.03-1.08), high HCT-CI score (OR, 1.12; 95% CI, 1.03-1.22), and not being in complete remission pre-HCT (OR, 1.74; 95% CI, 1.07-2.86) were independently associated with odds of having CHIP (eTable 4 in Supplement 1).

### CHIP and CVD Outcomes

**Figure 2.**
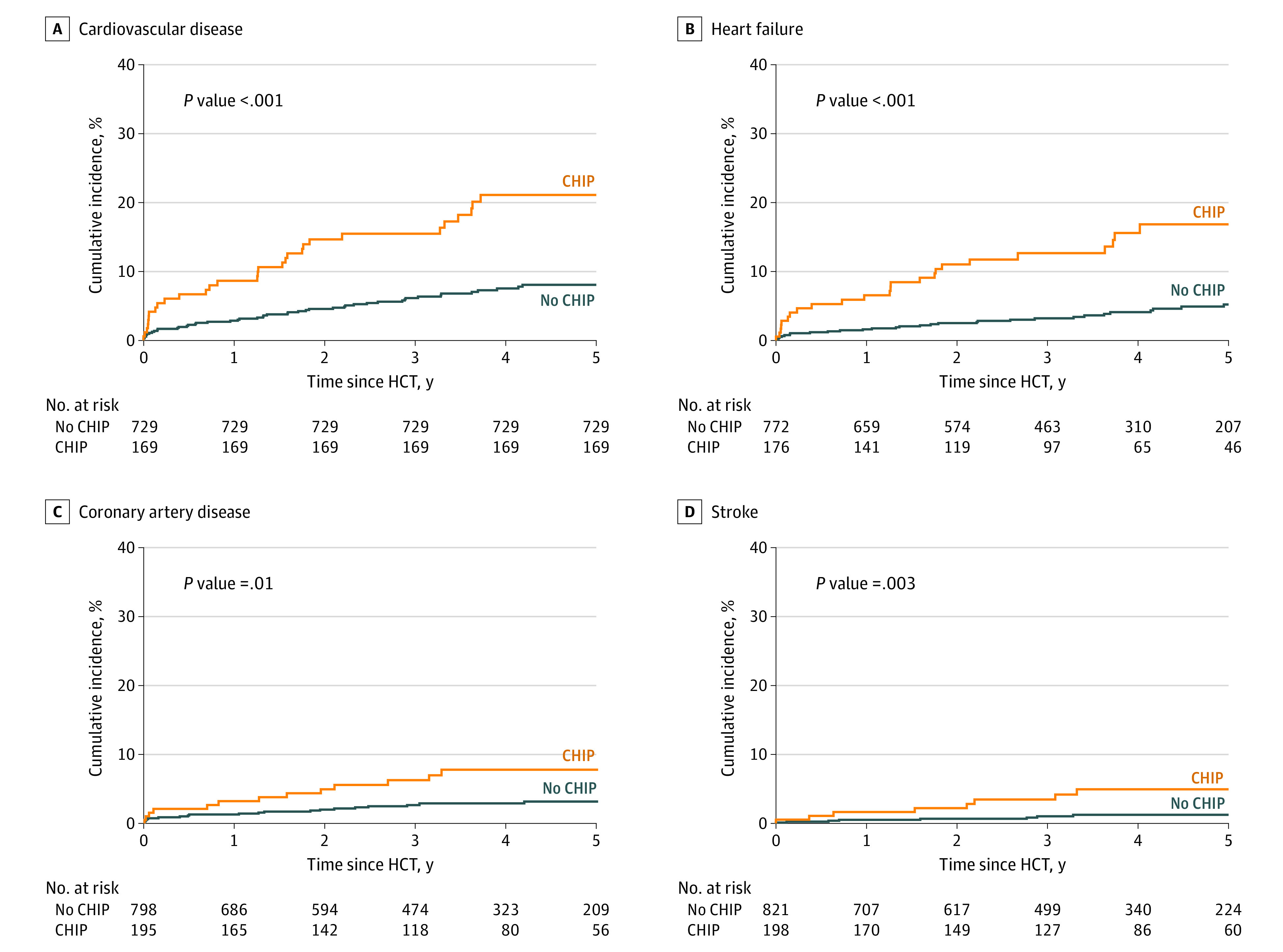
Five-Year Cumulative Incidence of Combined Cardiovascular Conditions CHIP indicates clonal hematopoiesis of indeterminate potential; HCT, hematopoietic cell transplant.

There were 79 patients who had 104 CVD events within 5 years from HCT; most CVD events were heart failure (54 [51.9%]) followed by CAD (34 [32.7%]) and stroke (16 [15.4%]). The 5-year cumulative incidence of a first CVD event was 10.8% and it was significantly higher among patients with CHIP compared with those without CHIP (21.1% vs 8.4%; *P* < .001; Figure 2A). The incidence of individual CVD outcomes was similarly higher among patients with CHIP compared with those without CHIP (Figure 2B, C, and D).

There was a graded association between the number of CHIP variants and the incidence of CVD by the following categories: no CHIP (8.39%), 1 CHIP variant (20.1%), and 2 or more variants (25.6%) (eFigure in Supplement 1). There was no significant association between CHIP VAF (2% to 5%, 5% to 10%, more than 10%) and CVD incidence (data not shown). Next, the study team evaluated gene-specific associations by examining the incidence of CVD according to the 3 most prevalent CHIP genes (*DNMT3A*, *TET2*, and *ASXL1*). Among patients with single CHIP mutations, the strongest association was observed with *ASXL1*, with 6 of 17 patients with the mutation developing CVD (36.4%) compared with 49 of 729 patients in those without CHIP (8.4%) (eTable 5 in Supplement 1). In subset analyses, *ASXL1* remained strongly associated with incidence of heart failure (37.7% vs 5.2%; *P* < .001), CAD (13.5% vs 3.2%; *P* = .01), and stroke (11.7% vs 1.2%; *P* < .001) (eTable 5 in Supplement 1).

**Figure 3.**
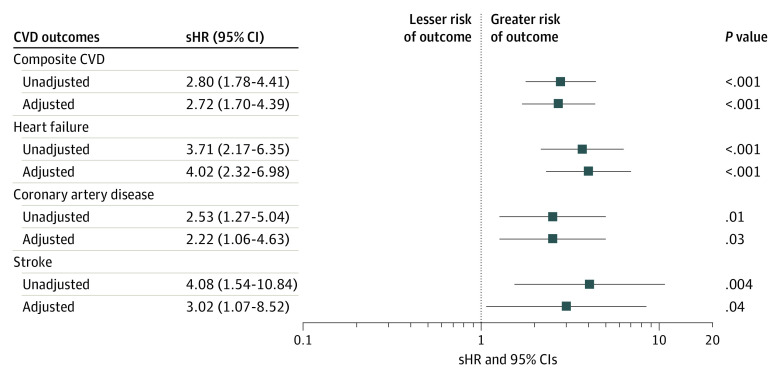
Association Between Clonal Hematopoiesis of Indeterminate Potential (CHIP) and Cardiovascular Disease (CVD) Outcomes Subdistrubtion hazard ratio (sHR) and 95% CIs for both combined and individual CVD outcomes adjusted for age, race and ethnicity, hematopoietic cell transplant-comorbidity index, hypertension, diabetes, dyslipidemia, and CHIP.

In univariable analysis, CHIP was associated with a greater than 2-fold risk of CVD (HR, 2.80; 95% CI, 1.78-4.41) at 5 years; additional significant risk factors for CVD included Black race (HR, 1.69; 95% CI, 1.00-2.86), older age at HCT (HR, 1.06; 95% CI, 1.02-1.10), high HCT-CI score (HR, 1.20; 95% CI, 1.07-1.33), hypertension (HR, 2.75; 95% CI, 1.64-4.61), and dyslipidemia (HR, 1.93; 95% CI, 1.24-3.00) (Figure 3; eTable 6 in Supplement 1). In the multivariable model, adjusting for age, race and ethnicity, HCT-CI, and hypertension, presence of CHIP at HCT was significantly and independently associated with risk of CVD (HR, 2.72; 95% CI, 1.69-4.39); sensitivity analysis censoring at the time of relapse yielded a similar magnitude of risk (HR, 2.52; 95% CI, 1.32-4.82). In subset analyses, CHIP was significantly associated with increased risk of heart failure (HR, 4.02; 95% CI, 2.32-6.98), CAD (HR, 2.22; 95% CI, 1.06-4.63), and stroke (HR, 3.02; 95% CI, 1.07-8.52) (Figure 2; eTable 7 in Supplement 1).

### Role of Modifiable Cardiovascular Risk Factors

The most prevalent cardiovascular risk factor at HCT was hypertension (56.7%), followed by dyslipidemia (42.4%) and diabetes (20.2%) (Table 1). There was a statistically significant incremental increase in the cumulative incidence of CVD by the following categories: no CHIP, no hypertension (4.1%); CHIP, no hypertension (12.0%); no CHIP, hypertension (11.8%); CHIP and hypertension (30.2%; *P* < .001) (Table 2). There was a similar trend in patients with dyslipidemia with the highest incidence observed in patients with CHIP and dyslipidemia (30.5%) (Table 2). In the multivariable regression models adjusting for age, race and ethnicity, HCT-CI score, and respective cardiovascular risk factors, the highest risks of CVD were observed among patients who had preexisting hypertension and CHIP (HR, 6.64; 95% CI, 2.97-14.83) followed by dyslipidemia and CHIP (HR, 3.87; 95% CI, 2.02-7.44) (Table 2). Of note, there was no statistically significant interaction between individual cardiovascular risk factors and CHIP in this analyses.

**Table 2.** Five-Year Cumulative Incidence and Risk of Cardiovascular Disease^a^

	No. of events/No. at risk	Cumulative incidence, % (95% CI)	Unadjusted		Adjusted	
			HR (95% CI)	*P* value	HR (95% CI)	*P* value
**Model 1**						
No hypertension						
No CHIP	11/327	4.1 (2.1-7.0)	1 [Reference]	NA	1 [Reference]	NA
CHIP	8/83	12.0 (5.4-21.5)	2.88 (1.16-7.17)	.02	2.45 (0.98-6.14)	.06
Hypertension						
No CHIP	38/402	11.8 (8.5-15.8)	2.86 (1.46-5.59)	.002	2.35 (1.16-4.76)	.02
CHIP	22/86	30.2 (19.8-41.3)	8.68 (4.21-17.90)	<.001	6.64 (2.97-14.83)	<.001
**Model 2**						
No dyslipidemia						
No CHIP	24/446	7.0 (4.5-10.1)	1 [Reference]	NA	1 [Reference]	NA
CHIP	11/95	13.5 (7.0-22.1)	2.23 (1.09-4.57)	.03	2.27 (1.10-4.70)	.03
Dyslipidemia						
No CHIP	25/283	10.6 (7.0-15.1)	1.64 (0.94-2.87)	.08	1.24 (0.71-2.19)	.45
CHIP	19/74	30.5 (19.2-42.6)	5.21 (2.87-9.46)	<.001	3.87 (2.02-7.44)	<.001
**Model 3**						
No diabetes						
No CHIP	36/597	7.5 (5.3-10.1)	1 [Reference]	NA	1 [Reference]	NA
CHIP	21/126	20.3 (12.9-28.8)	2.88 (1.68-3.94)	<.001	3.24 (1.87-5.60)	<.001
Diabetes						
No CHIP	13/132	12.3 (6.7-19.7)	1.6 (0.84-2.95)	.16	0.96 (0.50-1.81)	.90
CHIP	9/43	23.8 (11.5-38.5)	3.8 (1.80-7.83)	<.001	1.74 (0.83-3.66)	.15

Abbreviations: CHIP, clonal hematopoiesis of indeterminate potential, HR, hazard ratio; NA, not applicable.

^a^ All models were adjusted for age (continuous [years]), race and ethnicity (categorical: Black vs race other than Black), and hematopoietic cell transplant-comorbidity index (continuous integer). Additionally, model 1 was adjusted for dyslipidemia (yes/no) and diabetes (yes/no), model 2 was adjusted for hypertension (yes/no) and diabetes (yes/no), and model 3 was adjusted hypertension (yes/no) and dyslipidemia (yes/no).

## Discussion

In this study, leveraging a large, well-characterized, and demographically diverse cohort of patients with MM undergoing autologous HCT with available pre-HCT DNA, we found that CHIP was highly prevalent and associated with a significantly higher incidence of CVD after HCT compared with those without CHIP. In the adjusted model, CHIP was associated with a nearly 3-fold risk of developing any CVD and this association remained consistent by CVD subtype. The incidence of CVD after HCT was highest in patients with CHIP and hypertension or dyslipidemia (30% each), corresponding to nearly 7-fold and 4-fold increased risk of CVD, respectively, compared with those without CHIP and these modifiable risk factors. The findings from this study highlight the potential role of pre-HCT CHIP as a novel biomarker to better define CVD risk in patients with MM prior to HCT.

The current study is in line with the growing body of literature demonstrating that CHIP is a commonly occurring somatic mutation in patients with cancer exposed to cytotoxic therapies,^25,26^ given that all patients had received induction treatment prior to referral to HCT. That said, the associations between HCT-CI (comorbidity burden),^27^ complete remission status, and CHIP are unique. The association between high HCT-CI score and CHIP may be explained, in part, by the chronic proinflammatory state attributed to underlying comorbidities, an association also reported in patients living with HIV^28^ or those who develop premature menopause due to chronic illness.^29^ The association between complete remission status and CHIP may be attributed to a higher disease burden at HCT and associated challenges of maintaining durable response prior to HCT.

The presence of CHIP prior to HCT was significantly and independently associated with increased risk of de novo heart failure, CAD, stroke, as well as composite CVD. Sensitivity analysis censoring follow-up at the time of relapse/recurrence further confirmed these associations. There was also a graded association between the number of CHIP variants and incidence of CVD, suggesting a dose-dependent and additive effect. Variants in *ASXL1* were most significantly associated with risk of CVD. We speculate this association may be driven in part by that the high (more than 50%) proportion of CVD cases that were heart failure. *ASXL1* has been previously identified as uniquely associated with risk of having reduced left ventricular ejection fraction in a large nononcology population-based CHIP study^11^ and there is preclinical evidence to suggest a potential causative relationship between *ASXL1*-mediated clonal hematopoiesis and heart failure.^30^

Despite the strong association between CHIP and CVD, to our knowledge, to date, there are no CHIP-targeted interventions to modulate the associated CVD risk. Modifiable risk factors, such as hypertension, diabetes, and dyslipidemia, are established targets for CVD risk reduction in the general population but have not been studied in the context of CHIP. While there was no statistically significant interaction between these risk factors and CHIP, we believe our combined analyses reflect clinically meaningful subgroups of patients with MM. In our study, patients with CHIP and hypertension had nearly 7-fold risk of developing CVD and the risk was nearly 4-fold in patients with CHIP and dyslipidemia, compared with patients without CHIP and these individual risk factors. These results underscore the exceedingly high risk of CVD in the subset of patients with MM who have both CHIP and modifiable CVD risk factors, which may provide the rationale for resource allocation to develop more personalized interventions, such as early screening and treatment of these modifiable risk factors (eg, intensive blood pressure control and lipid-lowering strategies),^31^ in coordination with cardiologists to decrease the morbidity and mortality associated with CVD after HCT.

### Limitations

The findings from this study should be considered in the context of its limitations. First, we did not perform serial blood sampling after HCT to determine the course of CHIP variants after HCT. However, a recent study in nononcology patients who had targeted CHIP sequencing across a median span of 16 years showed that CHIP with VAF 2% or more tend to continue to expand over time, indicating that once CHIP is acquired, it is generally not lost.^32^ Second, while we did not find a significant association between pre-HCT MM treatments and either the prevalence of CHIP or incidence of post-HCT CVD in a subset of patients with available pre-HCT treatment data, we acknowledge that this lack of association may be due to heterogenous treatment exposures and relatively small subgroups in this cohort. Third, we did not have data regarding post-HCT therapies, such as immunomodulatory maintenance therapy (eg, lenalidomide), which could alter the underlying biology or inflammatory state and modulate subsequent cardiovascular risk. It was reassuring that censoring at relapse/progression did not alter the associations reported in the current study. Fourth, we acknowledge that despite limiting our genes of interest to leukemogenic variants, some may represent circulating tumor DNA from MM rather than CHIP. It is important to note that none of the commonly identified CHIP mutations in our study are hallmarks of MM^33^ and there was no association between complete remission status at the time of HCT and CVD risk after HCT. Lastly, we recognize the findings presented in this article will need to be validated in an independent cohort, which was outside the scope of our efforts.

## Conclusions

In summary, we demonstrate a significant association between CHIP and incident CVD after HCT in patients with MM, with the strongest association noted among patients undergoing HCT with modifiable risk factors, such as hypertension and diabetes. These findings may inform more targeted approaches to screening for CVD risk, allowing for improved CVD risk prediction prior to HCT and to guide the implementation of personalized preventive measures to improve health outcomes in at-risk patients. Additional studies are needed to further delineate if the association between CHIP and CVD exists in other cancer populations (eg, solid tumors, nonleukemic hematologic cancers) and to interrogate the specific gene-environment interactions that modulate long-term CVD risk.

## Notes

Supplement 1. eTable 1. List of CHIP-associated genes eTable 2. List of CHIP Variants eTable 3. Induction therapy regimens. Analysis limited to the subset of patients (n=666) with available pharmacy (A), as well as the entire cohort (N=1036), with unknown included as a categorical variable (B). eTable 4. Risk factors associated with CHIP eTable 5. Specific mutations and five-year cumulative incidence of CVD eTable 6. Univariable and multivariable analyses for risk of CVD after HCT eTable 7. Predictors of Individual Cardiovascular Disease Outcomes eFigure 1. Cumulative incidence of CVD by the number of mutations

Supplement 2. Data sharing statement
