# Supplementary material for: Clonal Hematopoiesis and Cardiovascular Disease in Patients With Multiple Myeloma Undergoing Hematopoietic Cell Transplant
Source: JAMA Cardiol. 2023 Nov 8;9(1):16–24. doi: 10.1001/jamacardio.2023.4105 (PMC10633387; doi:10.1001/jamacardio.2023.4105)
Supplement: Supplement 1. — eTable 1. List of CHIP-associated genes eTable 2. List of CHIP Variants eTable 3. Induction therapy regimens. Analysis limited to the subset of patients (n=666) with available pharmacy (A), as well as the entire cohort (N=1036), with unknown included as a categorical variable (B). eTable 4. Risk factors associated with CHIP eTable 5. Specific mutations and five-year cumulative incidence of CVD eTable 6. Univariable and multivariable analyses for risk of CVD after HCT eTable 7. Predictors of Individual Cardiovascular Disease Outcomes eFigure 1. Cumulative incidence of CVD by the number of mutations [file jamacardiol-e234105-s001.pdf]

Supplemental Online Content

Rhee JW, Pillai R, He T, et al. Clonal hematopoiesis and cardiovascular disease in patients with multiple myeloma undergoing hematopoietic cell transplant. *JAMA Cardiol*. Published online November 8, 2023. doi:10.1001/jamacardio.2023.4105

eTable 1. List of CHIP-associated genes
eTable 2. List of CHIP Variants
eTable 3. Induction therapy regimens. Analysis limited to the subset of patients (n=666) with available pharmacy (A), as well as the entire cohort (N=1036), with unknown included as a categorical variable (B).
eTable 4. Risk factors associated with CHIP
eTable 5. Specific mutations and five-year cumulative incidence of CVD
eTable 6. Univariable and multivariable analyses for risk of CVD after HCT
eTable 7. Predictors of Individual Cardiovascular Disease Outcomes
eFigure. Cumulative incidence of CVD by the number of mutations

This supplemental material has been provided by the authors to give readers additional information about their work.

© 2023 Rhee et al. *JAMA Cardiol.*

eTable 1. List of CHIP-associated genes

ANKRD26	*EP300*	*JAK3*	*PCCB*	*SMC3*
ASXL1	*ETNK1*	*KDM6A*	*PDS5B*	*SRCAP*
ASXL2	*ETV6*	*KIT*	*PDSS2*	*STAG1*
ATM	*EZH2*	*KLLN*	*PHF6*	*STAG2*
BCOR	*FBXO11*	*KMT2A*	*PHIP*	*STAT3*
BRAF	*FBXW7*	*KMT2B*	*PPM1D*	*SUZ12*
BRCA1	*FLT3*	*KMT2D*	*PRPF40B*	*TET2*
BRCA2	*FMNL3*	*KRAS*	*PRPF8*	*TP53*
BRCC3	*GATA1*	*LUC7L2*	*PTEN*	*U2AF1*
CBL	*GATA2*	*MFSD11*	*PTPN11*	*U2AF2*
CBLB	*GATA3*	*MIR636*	*RAD21*	*VPS33B*
CCDC157	*GNA13*	*MPL*	*RUNX1*	*WT1*
CEBPA	*GNAS*	*MSH6*	*SETBP1*	*YLPM1*
CHEK2	*GNB1*	*MYH88*	*SETD2*	*ZBTB33*
CREBBP	*HERC2*	*MYO6*	*SETDB1*	*ZNF318*
CSF1R	*IDH1*	*NF1*	*SF1*	*ZRSR2*
CSF3R	*IDH2*	*NOTCH2*	*SF3A1*	
CTC1	*IFZF3*	*NPM1*	*SF3B1*	
CTCF	*IKZF1*	*NR0B2*	*SH2B3*	
CUX1	*IKZF2*	*NRAS*	*SLC24A5*	
DARC	*IRAK1BP1*	*NXF1*	*SLC39A3*	
DNMT3A	*JAK1*	*PALB2*	*SLC45A2*	
EED	*JAK2*	*PAX5*	*SMC1A*	

© 2023 Rhee et al. *JAMA Cardiol.*

eTable 2. List of CHIP Variants

Gene	Chr	Position	Variant classification	Ref. Allele	Alt. Allele	Protein change	Ref. read	Alt. read	VAF
ASXL1	20	31021543	frameshift_variant	TG	-	p.Val515Glyfs*13	2268	799	26.05
ASXL1	20	31022442	frameshift_variant	-	G	p.Gly646Trpfs*12	1602	56	3.38
ASXL1	20	31022442	frameshift_variant	-	G	p.Gly646Trpfs*12	1269	29	2.23
ASXL1	20	31022793	stop_gained	C	T	p.Gln760Ter	1237	30	2.37
ASXL1	20	31022442	frameshift_variant	-	G	p.Gly646Trpfs*12	1597	33	2.02
ASXL1	20	31022442	frameshift_variant	-	G	p.Gly646Trpfs*12	1044	22	2.06
ASXL1	20	31022442	frameshift_variant	-	G	p.Gly646Trpfs*12	1509	33	2.14
ASXL1	20	31022442	frameshift_variant	-	G	p.Gly645Valfs*58	1040	26	2.44
ASXL1	20	31022442	frameshift_variant	-	G	p.Gly646Trpfs*12	1253	27	2.11
ASXL1	20	31022403	frameshift_variant	CACCACTGCCATAGAGAGGCGGC	-	p.Glu635Argfs*15	1801	50	2.70
ASXL1	20	31020689	missense_variant	T	G	p.Phe329Cys	404	314	43.73
ASXL1	20	31022442	frameshift_variant	-	G	p.Gly646Trpfs*12	937	48	4.87
ASXL1	20	31022442	frameshift_variant	-	G	p.Gly646Trpfs*12	1290	51	3.80
ASXL1	20	31022442	frameshift_variant	-	G	p.Gly646Trpfs*12	1020	23	2.21
ASXL1	20	31022442	frameshift_variant	-	G	p.Gly646Trpfs*12	1002	24	2.34
ASXL1	20	31022442	frameshift_variant	G	-	p.Gly645Valfs*58	1451	33	2.22
ASXL1	20	31022442	frameshift_variant	-	G	p.Gly646Trpfs*12	1732	40	2.26
ASXL1	20	31022937	frameshift_variant	C	-	p.Pro808Leufs*10	2307	241	9.46
ASXL1	20	31023213	frameshift_variant	CCCAT	-	p.Pro902Glufs*2	1877	69	3.55
ASXL1	20	31022442	frameshift_variant	-	G	p.Gly646Trpfs*12	2245	48	2.09
ASXL1	20	31022442	frameshift_variant	-	G	p.Gly646Trpfs*12	1703	37	2.13
ASXL1	20	31022442	frameshift_variant	-	G	p.Gly646Trpfs*12	1495	35	2.29
ASXL1	20	31022442	frameshift_variant	-	G	p.Gly646Trpfs*12	1643	66	3.86
ASXL1	20	31022442	frameshift_variant	-	G	p.Gly646Trpfs*12	1294	96	6.91
ASXL1	20	31022442	frameshift_variant	-	G	p.Gly646Trpfs*12	1466	36	2.40

© 2023 Rhee et al. *JAMA Cardiol.*

ASXL1	20	31022442	frameshift_variant	-	G	p.Gly646Trpfs*12	2536	60	2.31
ASXL1	20	31022442	frameshift_variant	-	G	p.Gly646Trpfs*12	1704	35	2.01
ASXL1	20	31022442	frameshift_variant	-	G	p.Gly646Trpfs*12	930	55	5.58
ASXL1	20	31022442	frameshift_variant	-	G	p.Gly646Trpfs*12	1600	54	3.26
ASXL1	20	31022442	frameshift_variant	-	G	p.Gly646Trpfs*12	1024	29	2.75
ASXL1	20	31022442	frameshift_variant	-	G	p.Gly646Trpfs*12	1616	34	2.06
ASXL1	20	31022442	frameshift_variant	-	G	p.Gly646Trpfs*12	1642	34	2.03
ASXL1	20	31022442	frameshift_variant	G	-	p.Gly645Valfs*58	1460	30	2.01
ASXL2	2	25991619	missense_variant	G	A	p.Ala208Val	3979	649	14.02
ASXL2	2	25965281	missense_variant	G	A	p.Pro1309Ser	271	6	2.17
ASXL2	2	25965281	missense_variant	G	A	p.Pro1309Ser	291	12	3.96
ASXL2	2	25966052	missense_variant	G	A	p.His1052Tyr	603	15	2.43
ASXL2	2	26022299	missense_variant	C	T	p.Gly120Ser	673	15	2.18
ASXL2	2	25965392	stop_gained	GC	AT	p.Gln1272Ter	1816	48	2.58
ATM	11	108175463	missense_variant	A	T	p.Asp1853Val	479	386	44.62
ATM	11	108139239	missense_variant	A	G	p.Asn914Ser	557	386	40.93
ATM	11	108200961	missense_variant	G	A	p.Arg2443Gln	1067	32	2.91
ATM	11	108160516	missense_variant	A	G	p.Tyr1475Cys	299	240	44.53
ATM	11	108172385	frameshift_variant	C	-	p.Arg1730Aspfs*8	956	34	3.43
ATM	11	108198384	missense_variant	C	G	p.Leu2330Val	770	329	29.94
ATM	11	108198384	missense_variant	C	G	p.Leu2330Val	994	26	2.55
ATM	11	108198384	missense_variant	C	G	p.Leu2330Val	865	112	11.46
ATM	11	108126976	missense_variant	G	A	p.Arg720His	1878	50	2.59
ATM	11	108138024	missense_variant	A	T	p.Ser865Cys	558	13	2.28
ATM	11	108196896	missense_variant	C	T	p.Leu2307Phe	1557	61	3.77
ATM	11	108196896	missense_variant	C	T	p.Leu2307Phe	948	539	36.25
BCOR	X	39932017	missense_variant	C	T	p.Arg861His	1272	39	2.97
BCOR	X	39922216	missense_variant	G	A	p.Ala1285Val	861	35	3.91

© 2023 Rhee et al. *JAMA Cardiol.*

BCOR	X	39934144	missense_variant	G	A	p.Pro152Leu	1054	30	2.77
BCOR	X	39933283	missense_variant	T	A	p.Lys439Met	996	22	2.16
BCOR	X	39934352	missense_variant	G	A	p.Arg83Trp	2442	76	3.02
BCOR	X	39913579	missense_variant	T	A	p.Leu1549Phe	302	8	2.58
BCOR	X	39923711	missense_variant	C	T	p.Ser1127Asn	296	7	2.31
BCOR	X	39914756	missense_variant	C	T	p.Asp1502Asn	336	9	2.61
BCOR	X	39916466	missense_variant	C	T	p.Val1479Met	216	9	4.00
BCORL1	X	129147895	missense_variant	G	C	p.Ala383Pro	445	354	44.31
BCORL1	X	129148457	missense_variant	C	T	p.Ala570Val	2037	47	2.26
BCORL1	X	129148289	missense_variant	C	T	p.Ser514Leu	856	24	2.73
BCORL1	X	129147500	missense_variant	C	T	p.Pro251Leu	439	10	2.23
BCORL1	X	129155098	missense_variant	G	A	p.Gly1194Arg	479	10	2.04
BCORL1	X	129147536	missense_variant	C	T	p.Pro263Leu	513	11	2.10
CBL	11	119148991	missense_variant	G	A	p.Cys404Tyr	547	12	2.15
CBL	11	119149238	missense_variant	T	C	p.Cys416Arg	1435	30	2.05
CBL	11	119149251	missense_variant	G	A	p.Arg420Gln	2255	91	3.88
CBL	11	119148874	splice_acceptor_variant	A	T		1013	29	2.78
CBL	11	119144697	missense_variant	C	T	p.Ser237Leu	1152	182	13.64
CBL	11	119149251	missense_variant	G	A	p.Arg420Gln	1248	52	4.00
CBL	11	119148982	missense_variant	G	A	p.Cys401Tyr	737	41	5.27
CSF1R	5	149449450	missense_variant	A	T	p.Ile499Lys	121	97	44.50
CSF1R	5	149447878	missense_variant	G	A	p.Pro509Leu	314	9	2.79
CSF1R	5	149456956	missense_variant	G	T	p.Gln258Lys	488	10	2.01
CSF3R	1	36945093	missense_variant	G	A	p.Ala2Val	740	17	2.25
DNMT3A	2	25470560	stop_gained	C	T	p.Trp305Ter	1750	36	2.02
DNMT3A	2	25467437	missense_variant	G	A	p.Leu547Phe	1753	36	2.01
DNMT3A	2	25464463	missense_variant	C	A	p.Val684Phe	981	28	2.78
DNMT3A	2	25467055	frameshift_variant	ATCT	-	p.Gln606Argfs*44	1104	38	3.33

© 2023 Rhee et al. *JAMA Cardiol.*

DNMT3A	2	25462077	missense_variant	G	C	p.Pro777Arg	783	165	17.41
DNMT3A	2	25457243	missense_variant	G	A	p.Arg882Cys	2314	640	21.67
DNMT3A	2	25457243	missense_variant	G	A	p.Arg882Cys	1307	693	34.65
DNMT3A	2	25467449	missense_variant	C	A	p.Gly543Cys	1853	141	7.07
DNMT3A	2	25457242	missense_variant	C	A	p.Arg882Leu	2039	55	2.63
DNMT3A	2	25463562	missense_variant	C	T	p.Gly707Asp	1009	64	5.96
DNMT3A	2	25469173	stop_gained	T	A	p.Lys429Ter	1055	518	32.93
DNMT3A	2	25457242	missense_variant	C	A	p.Arg882Leu	2542	61	2.34
DNMT3A	2	25463508	splice_donor_variant	C	T		1433	45	3.04
DNMT3A	2	25470498	missense_variant	G	A	p.Arg326Cys	1991	138	6.48
DNMT3A	2	25463541	missense_variant	G	C	p.Ser714Cys	912	87	8.71
DNMT3A	2	25467454	frameshift_variant	C	-	p.Cys541Leufs*110	1771	106	5.65
DNMT3A	2	25469096	frameshift_variant	-	G	p.Lys455Glnfs*18	4858	225	4.43
DNMT3A	2	25467448	missense_variant	C	G	p.Gly543Ala	1832	128	6.53
DNMT3A	2	25462068	missense_variant	A	G	p.Ile780Thr	952	62	6.11
DNMT3A	2	25463308	frameshift_variant	G	-	p.Arg729Glyfs*50	842	20	2.32
DNMT3A	2	25470516	stop_gained	G	A	p.Arg320Ter	2985	130	4.17
DNMT3A	2	25457242	missense_variant	C	A	p.Arg882Leu	1777	37	2.04
DNMT3A	2	25470914	stop_gained	C	A	p.Glu283Ter	2227	71	3.09
DNMT3A	2	25457290	splice_acceptor_variant	C	T		1102	126	10.26
DNMT3A	2	25467155	frameshift_variant	C	-	p.Ala574Glnfs*77	2182	103	4.51
DNMT3A	2	25470584	missense_variant	C	G	p.Trp297Ser	1495	81	5.14
DNMT3A	2	25457290	splice_acceptor_variant	C	T		676	87	11.40
DNMT3A	2	25464470	frameshift_variant	G	-	p.Met682Cysfs*23	1317	633	32.46
DNMT3A	2	25470535	stop_gained	C	T	p.Trp313Ter	2339	199	7.84
DNMT3A	2	25463576	missense_variant	A	T	p.Asp702Glu	1537	124	7.47
DNMT3A	2	25470497	missense_variant	C	T	p.Arg326His	2191	109	4.74
DNMT3A	2	25468186	missense_variant	C	T	p.Cys497Tyr	1577	45	2.77

© 2023 Rhee et al. *JAMA Cardiol.*

DNMT3A	2	25466797	missense_variant	C	T	p.Val636Met	3401	70	2.02
DNMT3A	2	25466799	missense_variant	C	T	p.Arg635Gln	2694	58	2.11
DNMT3A	2	25462086	splice_acceptor_variant	T	C		1468	98	6.26
DNMT3A	2	25463584	missense_variant	G	C	p.Pro700Ala	2292	60	2.55
DNMT3A	2	25468125	stop_gained	G	T	p.Cys517Ter	1159	175	13.12
DNMT3A	2	25467209	splice_acceptor_variant	T	C		1849	63	3.29
DNMT3A	2	25464487	missense_variant	G	A	p.Arg676Trp	2254	1413	38.53
DNMT3A	2	25464453	missense_variant	A	C	p.Val687Gly	1160	27	2.27
DNMT3A	2	25466851	frameshift_variant	C	-	p.Asp618Thrfs*33	1896	48	2.47
DNMT3A	2	25467472	missense_variant	G	T	p.Ser535Tyr	3331	94	2.74
DNMT3A	2	25469928	frameshift_variant	C	-	p.Val372Serfs*35	1433	38	2.58
DNMT3A	2	25467023	splice_donor_variant	C	A		2055	412	16.70
DNMT3A	2	25462006	missense_variant	T	C	p.Met801Val	2212	89	3.87
DNMT3A	2	25464487	missense_variant	G	A	p.Arg676Trp	4582	906	16.51
DNMT3A	2	25469139	stop_gained	C	T	p.Trp440Ter	2733	93	3.29
DNMT3A	2	25463307	missense_variant	C	A	p.Arg729Leu	1117	26	2.27
DNMT3A	2	25467132	stop_gained	C	T	p.Trp581Ter	2946	91	3.00
DNMT3A	2	25463237	missense_variant	G	T	p.Phe752Leu	2867	80	2.71
DNMT3A	2	25463191	missense_variant	C	T	p.Asp768Asn	1299	40	2.99
DNMT3A	2	25467071	stop_gained	GC	AT	p.Trp601Ter	2789	93	3.23
DNMT3A	2	25466788	missense_variant	G	A	p.Leu639Phe	1868	424	18.50
DNMT3A	2	25466800	missense_variant	G	A	p.Arg635Trp	3046	120	3.79
DNMT3A	2	25457243	missense_variant	G	A	p.Arg882Cys	3365	123	3.53
DNMT3A	2	25463211	frameshift_variant	AT	-	p.Met761Glyfs*3	2515	59	2.29
DNMT3A	2	25470020	frameshift_variant	AC	-	p.Val341Ter	956	25	2.55
DNMT3A	2	25470485	stop_gained	C	T	p.Trp330Ter	2960	62	2.05
DNMT3A	2	25464490	missense_variant	C	T	p.Val675Met	4077	150	3.55
DNMT3A	2	25457242	missense_variant	C	A	p.Arg882Leu	2127	186	8.04

© 2023 Rhee et al. *JAMA Cardiol.*

DNMT3A	2	25470494	stop_gained	C	T	p.Trp327Ter	2616	109	4.00
DNMT3A	2	25470992	frameshift_variant	T	AG	p.Thr257Leufs*7	1783	75	4.04
DNMT3A	2	25468202	splice_acceptor_variant	C	T		1596	51	3.10
DNMT3A	2	25462009	missense_variant	C	T	p.Gly800Ser	974	20	2.01
DNMT3A	2	25457242	missense_variant	C	A	p.Arg882Leu	2555	248	8.85
DNMT3A	2	25470905	splice_donor_variant	C	T		1934	43	2.18
DNMT3A	2	25462006	missense_variant	T	C	p.Met801Val	1180	38	3.12
DNMT3A	2	25467128	missense_variant	A	G	p.Cys583Arg	1468	34	2.26
DNMT3A	2	25464544	missense_variant	C	A	p.Val657Leu	1930	114	5.58
DNMT3A	2	25470497	missense_variant	C	T	p.Arg326His	743	594	44.43
DNMT3A	2	25467036	frameshift_variant	GTGGTTATTAGCGAAGAACATCT	-	p.Gln606Argfs*5	1446	56	3.73
DNMT3A	2	25462074	missense_variant	A	C	p.Val778Gly	1802	43	2.33
DNMT3A	2	25459804	splice_donor_variant	C	G		717	18	2.45
DNMT3A	2	25466799	missense_variant	C	T	p.Arg635Gln	2537	109	4.12
DNMT3A	2	25463515	frameshift_variant	G	-	p.Leu723Serfs*56	1597	49	2.98
DNMT3A	2	25463300	missense_variant	G	T	p.Phe731Leu	1758	72	3.93
DNMT3A	2	25469086	frameshift_variant	-	G	p.Arg458Profs*15	2416	203	7.75
DNMT3A	2	25469542	stop_gained	C	T	p.Trp409Ter	2034	48	2.31
DNMT3A	2	25467466	missense_variant	C	T	p.Cys537Tyr	1973	1097	35.73
DNMT3A	2	25463307	missense_variant	C	A	p.Arg729Leu	1418	29	2.00
DNMT3A	2	25470498	missense_variant	G	A	p.Arg326Cys	2714	65	2.34
DNMT3A	2	25458688	splice_acceptor_variant	TGCTGAACTAGATGAAGAGG	C		862	30	3.36
DNMT3A	2	25468925	frameshift_variant	C	-	p.Val480Cysfs*171	1708	42	2.40
DNMT3A	2	25464520	missense_variant	C	T	p.Val665Met	2198	398	15.33
DNMT3A	2	25463235	stop_gained	C	T	p.Trp753Ter	3762	259	6.44
DNMT3A	2	25457257	frameshift_variant	-	A	p.Ser878Leufs	1852	76	3.94
DNMT3A	2	25457243	missense_variant	G	A	p.Arg882Cys	1283	492	27.72
DNMT3A	2	25463508	splice_donor_variant	C	T		1292	38	2.86

© 2023 Rhee et al. *JAMA Cardiol.*

DNMT3A	2	25457243	missense_variant	G	A	p.Arg882Cys	2046	75	3.54
DNMT3A	2	25467448	missense_variant	G	C	p.Gly543Ala	1891	60	3.08
DNMT3A	2	25467484	missense_variant	T	A	p.Asp531Val	2307	222	8.78
DNMT3A	2	25464487	missense_variant	G	A	p.Arg676Trp	2483	227	8.38
DNMT3A	2	25463248	missense_variant	G	A	p.Arg749Cys	3260	128	3.78
DNMT3A	2	25469544	frameshift_variant	TTCAATCATGGGC	-	p.Lys404Asnfs*243	2036	92	4.32
DNMT3A	2	25463287	frameshift_variant	G	-	p.Arg736Alafs*43	4028	138	3.31
DNMT3A	2	25470535	stop_gained	C	T	p.Trp313Ter	2743	409	12.98
DNMT3A	2	25463587	missense_variant	C	T	p.Gly699Ser	1507	61	3.89
DNMT3A	2	25463212	missense_variant	T	C	p.Met761Val	3221	133	3.97
DNMT3A	2	25466836	frameshift_variant	A	-	p.Tyr623Thrfs*28	989	45	4.35
DNMT3A	2	25458696	splice_acceptor_variant	T	C		811	24	2.87
DNMT3A	2	25467191	missense_variant	A	C	p.Cys562Gly	1576	48	2.96
DNMT3A	2	25457243	missense_variant	G	A	p.Arg882Cys	1283	492	27.72
DNMT3A	2	25464487	missense_variant	G	A	p.Arg676Trp	2483	227	8.38
DNMT3A	2	25457242	missense_variant	C	A	p.Arg882Leu	2533	82	3.14
DNMT3A	2	25457242	missense_variant	C	A	p.Arg882Leu	2533	82	3.14
GNAS	20	57484420	missense_variant	C	T	p.Arg201Cys	969	25	2.52
GNAS	20	57484421	missense_variant	G	A	p.Arg201His	1412	34	2.35
GNAS	20	57484421	missense_variant	G	A	p.Arg201His	1392	56	3.87
GNB1	1	1747229	missense_variant	T	C	p.Lys57Glu	1743	191	9.88
GNB1	1	1747229	missense_variant	T	C	p.Lys57Glu	4255	147	3.34
GNB1	1	1747229	missense_variant	T	C	p.Lys57Glu	2729	70	2.50
GNB1	1	1747229	missense_variant	T	C	p.Lys57Glu	2647	331	11.11
GNB1	1	1747229	missense_variant	T	C	p.Lys57Glu	2777	81	2.83
GNB1	1	1747229	missense_variant	T	C	p.Lys57Glu	1985	434	17.94
GNB1	1	1747229	missense_variant	T	C	p.Lys57Glu	1646	904	35.45
GNB1	1	1747229	missense_variant	T	C	p.Lys57Glu	1822	235	11.42

© 2023 Rhee et al. *JAMA Cardiol.*

KRAS	12	25398284	missense_variant	C	T	p.Gly12Asp	1760	36	2.00
NPM1	5	170819969	inframe_deletion	GAT	-	p.Asp173del	1386	586	29.72
PHF6	X	133511733	missense_variant	G	A	p.Gly29Glu	2110	183	7.98
PHF6	X	133527965	missense_variant	C	T	p.Thr134Ile	201	7	3.37
RUNX1	21	36164772	inframe_insertion	-	ATGCCG	p.Ile366_Gly367dup	626	405	39.28
SF3B1	2	198266834	missense_variant	T	C	p.Lys700Glu	1973	405	17.03
TET2	4	106157069	stop_gained	C	A	p.Ser657Ter	887	132	12.95
TET2	4	106157329	stop_gained	C	T	p.Gln744Ter	1601	45	2.73
TET2	4	106193850	frameshift_variant	-	A	p.Arg1440Thrfs*38	1690	145	7.90
TET2	4	106190831	missense_variant	G	A	p.Gly1370Glu	1190	29	2.38
TET2	4	106164853	missense_variant	G	C	p.Ala1241Pro	2127	50	2.30
TET2	4	106155939	frameshift_variant	-	T	p.Asn281Ter	949	120	11.23
TET2	4	106157560	stop_gained	C	T	p.Gln821Ter	1531	141	8.43
TET2	4	106157252	frameshift_variant	T	-	p.Leu719Cysfs*32	820	22	2.61
TET2	4	106155436	frameshift_variant	A	-	p.Lys113Asnfs*15	2234	76	3.29
TET2	4	106197317	missense_variant	A	G	p.Thr1884Ala	1259	48	3.67
TET2	4	106197181	frameshift_variant	-	TGGTG	p.Ala1840Valfs*49	2581	80	3.01
TET2	4	106156729	stop_gained	C	T	p.Arg544Ter	2631	84	3.09
TET2	4	106194059	frameshift_variant	G	-	p.Ala1508Leufs*63	2257	81	3.46
TET2	4	106196233	frameshift_variant	-	G	p.Gln1523Alafs*55	2196	170	7.19
TET2	4	106190861	missense_variant	A	G	p.His1380Arg	1153	29	2.45
TET2	4	106164824	missense_variant	T	C	p.Leu1231Pro	3162	224	6.62
TET2	4	106197269	missense_variant	C	T	p.His1868Tyr	2714	68	2.44
TET2	4	106158208	frameshift_variant	-	GCGT	p.Lys1038Cysfs*6	1449	55	3.66
TET2	4	106156747	stop_gained	C	T	p.Arg550Ter	3680	445	10.79
TET2	4	106197317	missense_variant	A	G	p.Thr1884Ala	1599	75	4.48
TET2	4	106180839	missense_variant	T	G	p.Cys1289Trp	1188	29	2.38
TET2	4	106194075	missense_variant	G	A	p.Glu1513Lys	1290	56	4.16

© 2023 Rhee et al. *JAMA Cardiol.*

TET2	4	106157197	stop_gained	A	T	p.Lys700Ter	1114	92	7.63
TET2	4	106197285	missense_variant	T	C	p.Ile1873Thr	998	55	5.22
TET2	4	106164913	missense_variant	C	T	p.Arg1261Cys	1577	35	2.17
TET2	4	106157126	frameshift_variant	-	TG	p.Gly678Valfs*23	1745	98	5.32
TET2	4	106183003	missense_variant	C	A	p.Gln1348Lys	2229	66	2.88
TET2	4	106197317	missense_variant	A	G	p.Thr1884Ala	1842	55	2.90
TET2	4	106164840	frameshift_variant	C	-	p.Pro1237Argfs*16	1867	40	2.10
TET2	4	106180792	stop_gained	C	T	p.Gln1274Ter	618	260	29.61
TET2	4	106157422	frameshift_variant	-	T	p.Phe776Leufs*5	1846	612	24.90
TET2	4	106197186	missense_variant	C	T	p.Ala1840Val	2544	82	3.12
TET2	4	106158430	frameshift_variant	-	T	p.Leu1111Phefs*19	2000	110	5.21
TET2	4	106196592	frameshift_variant	G	-	p.Cys1642Serfs*53	2805	223	7.36
TET2	4	106156951	stop_gained	C	T	p.Gln618Ter	4377	1114	20.29
TET2	4	106156729	stop_gained	C	T	p.Arg544Ter	2046	93	4.35
TET2	4	106158372	frameshift_variant	ACCA	-	p.Thr1093Lysfs*12	1274	29	2.23
TET2	4	106180870	missense_variant	T	G	p.Phe1300Val	1556	39	2.45
TET2	4	106194073	missense_variant	C	T	p.Ala1512Val	1890	79	4.01
TET2	4	106182948	frameshift_variant	GTCCACTCTTA	-	p.Ser1330Glyfs*5	1876	265	12.38
TET2	4	106196705	stop_gained	C	T	p.Gln1680Ter	920	33	3.46
TP53	17	7578272	missense_variant	G	A	p.His193Tyr	2192	49	2.19
TP53	17	7576897	stop_gained	G	A	p.Gln317Ter	1235	39	3.06
TP53	17	7577529	missense_variant	A	C	p.Ile251Ser	678	25	3.56
TP53	17	7577563	missense_variant	T	A	p.Ser240Cys	936	37	3.80
TP53	17	7577097	missense_variant	C	G	p.Asp281His	1776	319	15.23
TP53	17	7577539	missense_variant	G	A	p.Arg248Trp	941	290	23.56
TP53	17	7577568	missense_variant	C	A	p.Cys238Phe	1079	117	9.78
TP53	17	7579329	missense_variant	T	C	p.Lys120Glu	2193	81	3.56
TP53	17	7578407	missense_variant	G	C	p.Arg175Gly	2130	88	3.97

© 2023 Rhee et al. *JAMA Cardiol.*

TP53	17	7578406	missense_variant	C	T	p.Arg175His	1965	137	6.52
TP53	17	7578415	missense_variant	A	T	p.Val172Asp	2476	88	3.43
TP53	17	7578394	missense_variant	T	C	p.His179Arg	1015	85	7.73
TP53	17	7578513	missense_variant	C	G	p.Lys139Asn	1697	54	3.08
TP53	17	7577124	missense_variant	C	T	p.Val272Met	1952	96	4.69
TP53	17	7577100	missense_variant	T	C	p.Arg280Gly	1660	585	26.06
TP53	17	7577121	missense_variant	G	A	p.Arg273Cys	823	452	35.45
TP53	17	7577120	missense_variant	C	T	p.Arg273His	1707	40	2.29
TP53	17	7578509	missense_variant	A	G	p.Cys141Arg	915	72	7.29
TP53	17	7579592	splice_acceptor_variant	TGTA	-		1717	62	3.49

© 2023 Rhee et al. *JAMA Cardiol.*

eTable 3. Induction therapy regimens. Analysis limited to the subset of patients (n=666) with available pharmacy (A), as well as the entire cohort (N=1036), with unknown included as a categorical variable (B).

(A)

**Induction Therapy Regimen**	**Total (n=666) No. (%)**	**CHIP (n= 127) No. (%)**	**No CHIP (n= 539) No. (%)**	***P*-value**
Cyclophosphamide, Bortezomib, Dexamethasone	108 (16.2)	16 (12.6)	92 (17.1)	0.71
Cyclophosphamide, Lenalidomide, Bortezomib, Dexamethasone	86 (12.9)	18 (14.2)	68 (12.6)	
Lenalidomide, Bortezomib, Dexamethasone	322 (48.3)	67 (52.8)	255 (47.3)	
Lenalidomide, Dexamethasone	66 (9.9)	10 (7.9)	56 (10.4)	
Bortezomib, Dexamethasone	80 (12.0)	15 (11.8)	65 (12.1)	
Other	4 (0.6)	1 (0.8)	3 (0.6)	

(B)

**Induction Therapy Regimen**	**Total (n=1036) No. (%)**	**CHIP (n= 201) No. (%)**	**No CHIP (n= 835) No. (%)**	***P*-value**
Cyclophosphamide, Bortezomib, Dexamethasone	108 (10.4)	16 (8.0)	92 (11.0)	0.80
Cyclophosphamide, Lenalidomide, Bortezomib, Dexamethasone	86 (8.3)	18 (9.0)	68 (8.1)	
Lenalidomide, Bortezomib, Dexamethasone	322 (31.1)	67 (33.3)	255 (30.5)	
Lenalidomide, Dexamethasone	66 (6.4)	10 (5.0)	56 (6.7)	
Bortezomib, Dexamethasone	80 (7.7)	15 (7.5)	65 (7.8)	
Other	4 (0.4)	1 (0.5)	3 (0.4)	
Unknown	370 (35.7)	74 (36.8)	296 (35.5)	

Abbreviations: CHIP, clonal hematopoiesis of indeterminate potential.

© 2023 Rhee et al. *JAMA Cardiol.*

eTable 4. Risk factors associated with CHIP

Variable	Univariable Analysis		Multivariable Analysis	
	**OR (95% CI)**	***P*-value**	**OR (95% CI)**	***P*-value**
Age	1.06 (1.04-1.08)	<0.001	1.05 (1.03-1.08)	<0.001
Gender				
Female	1.00 (Referent)	–		
Male	1.01 (0.74-1.38)	0.898		
Race				
Other*	1.00 (Referent)	–		
Black race	0.70 (0.44-1.12)	0.137		
BMI	0.97 (0.94-1.00)	0.065	0.97 (0.94-1.00)	0.062
HCT-CI	1.15 (1.06-1.25)	<0.001	1.12 (1.03-1.22)	0.007
KPS	0.99 (0.98-1.01)	0.389		
Remission status at HCT				
CR	1.00 (Referent)	–	1.00 (Referent)	–
Not in CR	1.86 (1.15-3.02)	0.012	1.74 (1.07-2.86)	0.027
Hypertension status				
No	1.00 (Referent)	–		
Yes	0.89 (0.65-1.21)	0.439		
Diabetes status				
No	1.00 (Referent)	–	1.00 (Referent)	–
Yes	1.56 (1.09-2.23)	0.015	1.44 (0.98-2.11)	0.065
Dyslipidemia status				
No	1.00 (Referent)	–		
Yes	1.22 (0.89-1.66)	0.214		

Abbreviation: CHIP, clonal hematopoiesis of indeterminate potential; OR, odds ratio; CI, confidence interval; BMI, body mass index; HCT-CI, hematopoietic cell transplantation-comorbidity index; KPS, Karnofsky performance scale; CR, complete remission.
* Race and ethnicity other than Black race

© 2023 Rhee et al. *JAMA Cardiol.*

eTable 5. Specific mutations and five-year cumulative incidence of CVD*

Specific single gene mutations	Reference group	Cumulative incidence of CVD	*P*-value
**Composite CVD**			
DNMT3A mutations alone (n=59)	No mutations (n=729)	16.8% vs. 8.4%	0.050
TET2 mutations alone (n=24)	No mutations (n=729)	20.3% vs. 8.4%	0.069
ASXL1 mutations alone (n=17)	No mutations (n=729)	36.4% vs. 8.4%	<0.001
**Heart failure**			
DNMT3A mutations alone (n=62)	No mutations (n=772)	11.8% vs. 5.2%	0.024
TET2 mutations alone (n=24)	No mutations (n=772)	15.6% vs. 5.2%	0.041
ASXL1 mutations alone (n=18)	No mutations (n=772)	37.7% vs. 5.2%	<0.001
**Coronary artery disease**			
DNMT3A mutations alone (n=67)	No mutations (n=798)	7.2% vs. 3.2%	0.116
TET2 mutations alone (n=27)	No mutations (n=798)	4.4% vs. 3.2%	0.770
ASXL1 mutations alone (n=24)	No mutations (n=798)	13.5% vs. 3.2%	0.009
**Stroke**			
DNMT3A mutations alone (n=70)	No mutations (n=821)	5.8% vs. 1.2%	0.015
TET2 mutations alone (n=27)	No mutations (n=821)	4.8% vs. 1.2%	0.210
ASXL1 mutations alone (n=26)	No mutations (n=821)	11.7% vs. 1.2%	<0.001

*Differences in numbers among the CVD and reference groups reflects censoring based on outcomes of interest.
Abbreviations: CVD, cardiovascular disease.

© 2023 Rhee et al. *JAMA Cardiol.*

eTable 6. Univariable and multivariable analyses for risk of CVD after HCT

Variable	Combined CVD<br>Univariable Analysis<br>sHR (95% CI)	*P*-value	Multivariable Analysis<br>sHR (95% CI)	*P*-value
CHIP				
No	1.00 (Referent)	–	1.00 (Referent)	–
Yes	2.80 (1.78-4.41)	<0.001	2.72 (1.69-4.39)	<0.001
Age	1.06 (1.02-1.10)	0.003	1.04 (1.00-1.08)	0.086
Gender				
Female	1.00 (Referent)	–		
Male	1.17 (0.75-1.83)	0.484		
Race				
Other*	1.00 (Referent)	–	1.00 (Referent)	–
Black race	1.69 (1.00-2.86)	0.052	1.66 (0.96-2.87)	0.071
BMI	1.02 (0.98-1.07)	0.309		
HCT-CI	1.20 (1.07-1.33)	0.001	1.11 (1.01-1.22)	0.029
Remission status at HCT				
CR	1.00 (Referent)	–		
Not in CR	1.07 (0.58-1.97)	0.823		
Hypertension status				
No	1.00 (Referent)	–	1.00 (Referent)	–
Yes	2.75 (1.64-4.61)	<0.001	2.49 (1.42-4.38)	0.002
Diabetes status				
No	1.00 (Referent)	–	1.00 (Referent)	–
Yes	1.57 (0.96-2.55)	0.073	0.76 (0.44-1.32)	0.331
Dyslipidemia status				
No	1.00 (Referent)	–	1.00 (Referent)	–
Yes	1.93 (1.24-3.00)	0.004	1.39 (0.87-2.22)	0.164

Abbreviation: CHIP, clonal hematopoiesis of indeterminate potential; CR, complete remission; OR, odds ratio; BMI, body mass index; HCT-CI, hematopoietic cell transplantation-comorbidity index; KPS, Karnofsky performance scale.
* Race and ethnicity other than Black race

© 2023 Rhee et al. *JAMA Cardiol.*

eTable 7. Predictors of Individual Cardiovascular Disease Outcomes

Variable	Heart Failure				Coronary Artery Disease				Stroke			
	Univariable Analysis		Multivariable Analysis		Univariable Analysis		Multivariable Analysis		Univariable Analysis		Multivariable Analysis	
	sHR (95% CI)	*P*-value	sHR (95% CI)	*P*-value	sHR (95% CI)	*P*-value	sHR (95% CI)	*P*-value	sHR (95% CI)	*P*-value	sHR (95% CI)	*P*-value
CHIP												
No	1.00 (Referent)	–	1.00 (Referent)	–	1.00 (Referent)	–	1.00 (Referent)	–	1.00 (Referent)	–	1.00 (Referent)	–
Yes	3.71 (2.17-6.35)	<0.001	4.02 (2.32-6.98)	<0.001	2.53 (1.27-5.04)	0.008	2.22 (1.06-4.63)	0.034	4.08 (1.54-10.84)	0.005	3.02 (1.07-8.52)	0.037
Age	1.04 (1.00-1.09)	0.056	1.02 (0.97-1.07)	0.390	1.07 (1.02-1.13)	0.007	1.05 (0.99-1.11)	0.084	1.10 (1.02-1.18)	0.017	1.07 (0.99-1.15)	0.085
Gender												
Female	1.00 (Referent)	–			1.00 (Referent)	–			1.00 (Referent)	–		
Male	1.06 (0.62-1.82)	0.824			1.51 (0.75-3.05)	0.252			1.01 (0.38-2.73)	0.978		
Race												
Race*	1.00 (Referent)	–	1.00 (Referent)	–			1.00 (Referent)	–	1.00 (Referent)	–	1.00 (Referent)	–
Black race	1.97 (1.08-3.61)	0.028	2.15 (1.16-3.97)	0.015	1.40 (0.61-3.23)	0.426	1.30 (0.56-3.03)	0.544	0.73 (0.18-3.40)	0.733	0.60 (0.13-2.87)	0.524
BMI	1.02 (0.96-1.07)	0.588			0.99 (0.94-1.04)	0.664			1.05 (0.98-1.12)	0.152	–	–
HCT-CI	1.15 (1.02-1.29)	0.027	1.09 (0.97-1.22)	0.149	1.15 (0.95-1.40)	0.119	1.05 (0.87-1.26)	0.618	1.17 (0.99-1.40)	0.070	1.01 (0.86-1.18)	0.913
KPS	1.00 (0.97-1.03)	0.795			1.01 (0.97-1.05)	0.894			1.00 (0.94-1.07)	0.912	–	–
Complete Remission												
Yes	1.00 (Referent)	–			1.00 (Referent)	–			1.00 (Referent)	–		
No	1.28 (0.58-2.82)	0.546			1.12 (0.43-2.88)	0.818			2.84 (0.38-21.41)	0.312		
Hypertension status												
No	1.00 (Referent)	–	1.00 (Referent)	–	1.00 (Referent)	–	1.00 (Referent)	–	1.00 (Referent)	–	1.00 (Referent)	–
Yes	2.32 (1.26-4.26)	0.007	2.42 (1.25-4.70)	0.009	2.60 (1.18-5.74)	0.018	2.15 (0.91-5.08)	0.080	2.33 (0.75-7.22)	0.144	1.41 (0.41-4.85)	0.588
Diabetes status												
No	1.00 (Referent)	–	1.00 (Referent)	–	1.00 (Referent)	–	1.00 (Referent)	–			1.00 (Referent)	–
Yes	1.14 (0.60-2.16)	0.687	0.57 (0.29-1.14)	0.110	1.59 (0.76-3.32)	0.218	0.81 (0.37-1.75)	0.587	3.86 (1.45-10.26)	0.007	2.56 (0.76-8.61)	0.130
Dyslipidemia status												
No	1.00 (Referent)	–	1.00 (Referent)	–	1.00 (Referent)	–	1.00 (Referent)	–	1.00 (Referent)	–	1.00 (Referent)	–
Yes	1.43 (0.84-2.43)	0.189	1.11 (0.65-1.91)	0.702	2.58 (1.28-5.22)	0.008	1.88 (0.86-4.11)	0.113	2.96 (1.03-8.05)	0.044	1.65 (0.54-5.08)	0.382

Abbreviation: CI, confidence interval; CHIP, clonal hematopoiesis of indeterminate potential; BMI, body mass index; HCT-CI, hematopoietic cell transplantation-comorbidity index; KPS, Karnofsky performance scale, sHR, subdistribution hazard ratio.
The multivariable models were included: age, race/ethnicity, HCT-CI, hypertension, diabetes, dyslipidemia, and CHIP.
* Race and ethnicity other than Black race

© 2023 Rhee et al. *JAMA Cardiol.*

© 2023 Rhee et al. *JAMA Cardiol.*

eFigure 1. Cumulative incidence of CVD by the number of mutations. The color of the lines indicates cumulative incidence of CVD among patients without clonal hematopoiesis of indeterminate potential (CHIP) (grey), patients with one CHIP mutation (red), and patients with more than one CHIP mutation (blue).

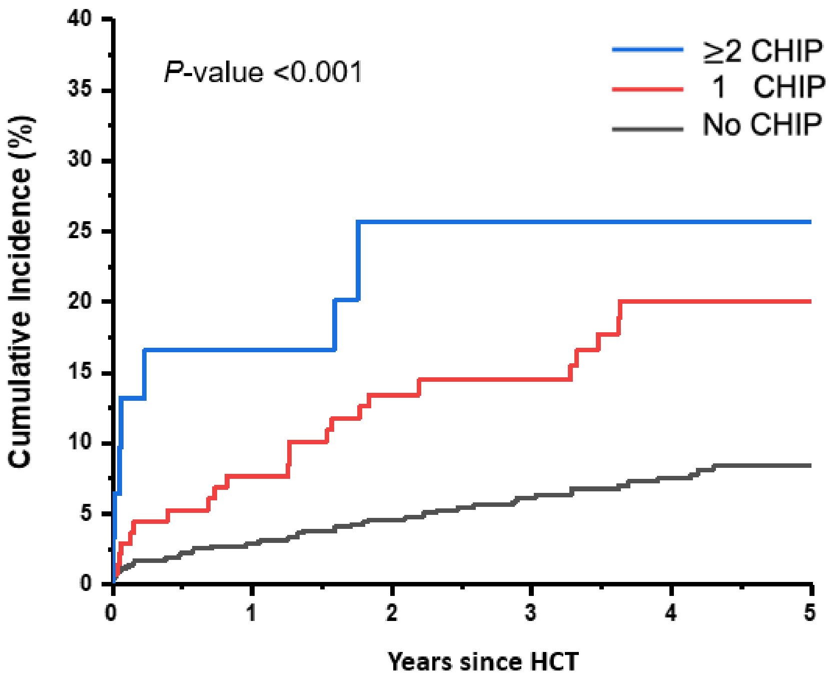

© 2023 Rhee et al. *JAMA Cardiol.*
