# Supplementary material for: Clonal Hematopoiesis and Cardiovascular Disease in Patients With Multiple Myeloma Undergoing Hematopoietic Cell Transplant
Source: JAMA Cardiol. 2023 Nov 8;9(1):16–24. doi: 10.1001/jamacardio.2023.4105 (PMC10633387; doi:10.1001/jamacardio.2023.4105)
Supplement: Supplement 2. — Data sharing statement [file jamacardiol-e234105-s002.pdf]

Data Sharing Statement

Data
Data available: Yes
Data types: Deidentified participant data
How to access data: Data will be available upon request (please email: SArmenian@coh.org) once final analyses are complete
When available: With publication

Supporting Documents
Document types: None

Additional Information
Who can access the data: anyone requesting the data
Types of analyses: for research purposes
Mechanisms of data availability: with investigator support
